# Perspective on Antibiotic Resistance in Bangladesh: A Critical Yet Overlooked Public Health Crisis

**DOI:** 10.1002/hsr2.70407

**Published:** 2025-01-24

**Authors:** Md. Mahadi Hassan, Noushin Nohor

**Affiliations:** ^1^ Department of Public Health and Informatics Jahangirnagar University Savar Dhaka Bangladesh; ^2^ Health Data Science Lab, Center for Health Innovation, Research Action, and Learning—Bangladesh (CHIRAL Bangladesh) Dhanmondi Dhaka Bangladesh

**Keywords:** antibiotic resistance, antibiotics, antimicrobial resistance

## Abstract

**Background and Aims:**

This article explores antibiotic resistance as a critical public health issue in Bangladesh, emphasizing its growing threat to the nation's healthcare system. As a developing country, Bangladesh faces unique challenges in managing this crisis, with antibiotic resistance posing significant risks due to its multidimensional problems to both individual health and the broader population.

**Methods:**

We searched for relevant pieces of literature that discuss the antibiotic resistance problem both from a global and national perspective. Based on the importance and our objectives we selected the relevant articles. The perspective was written using the evidence and information available in those articles.

**Results:**

Key factors contributing to the rise of antibiotic resistance include the irrational use and prescription of antibiotics in both healthcare settings and agriculture, incomplete doses of antibiotics, and insufficient public awareness. The article also discusses potential policy recommendations, such as the implementation of stricter antimicrobial stewardship programs, enhanced surveillance systems, and public education campaigns to reduce the misuse of antibiotics.

**Conclusion:**

In conclusion, antibiotic resistance in Bangladesh requires urgent attention and coordinated efforts from all sectors of society to prevent further deterioration of public health and safeguard future generations.

## 1 Background

Antibiotic Resistance in Bangladesh is a crucial public health concern, which has been left unattended for a long period in the country. Antibiotic resistance means, the status of a bacteria to gain immunity against one or more traditional antibiotics. Bacteria can acquire antibiotic resistance through different mechanisms—mutational adaptations, acquiring genetic materials, and alteration of gene expression, all of which can make them resistant to traditional antibiotics. It can resist the effect of antibiotics by—preventing or changing drug targets, and directly modifying or, inactivating antibiotics. Antibiotic resistance is usually categorized into three types based on the mechanism of resistance of a bacterium—(i) intracellular antibiotic concentration reduction, (ii) antibiotic target modification, and (iii) antibiotic inactivation [1, 2]. However, there could be several other mechanisms through which a bacterium can achieve antibiotic resistance.

Due to the increasing prevalence of antibiotic resistance, and the decreasing amount of new antibiotic inventions, antibiotic resistance has become a major public health concern from a global perspective [3]. This has now resulted in a higher probability of treatment failure, relapsing infections, elevated risk and prevalence of mortality, morbidity, and increased healthcare expenditure [4, 5]. In 2019, antibiotic resistance was said to be directly responsible for around 1.27 million global deaths, and in total contributed to about 5 million global deaths, which is higher than the deaths due to malaria, AIDS, and so forth [6]. It is also estimated that antimicrobial resistance could be involved in approximately 10 million deaths annually by 2050. The expected global economic burden is projected over 100 trillion USD [7].

## 2 The Bangladesh Context of Antibiotic Resistance

Developing countries are at greater risk due to antibiotic resistance and the severity of the consequences faced by them is far more than the developed countries, due to the already existing health disparities and problems such as poor knowledge and awareness, limited laws and regulations regarding antibiotics, poor law enforcement, poor health‐seeking behaviors, misuse of antibiotics, and inadequate surveillance [8]. Bangladesh is not an exception in this regard. National Antimicrobial Resistance Surveillance, Bangladesh, 2016–2023 revealed that Antibiotic resistance in Bangladesh has increased by 11% over the past 5 years, at the same time, the effectiveness of several commonly used antibiotics decreased by up to 82% in 2023, compared to 71% in 2016 [9]. This is also evident in a systematic review conducted on a total of 46 papers, which examined the current situation of antibiotic resistance in Bangladesh. They synthesized evidence of many bacteria such as *Escherichia coli*, *Klebsiella* spp., *Pseudomonas* spp., *Staphylococcus aureus*, *Salmonella* spp., *Acinetobacter* spp., *Enterococcus* spp., *Streptococcus pneumoniae*, *Shigella* spp., *Enterobacter* spp., *Helicobacter pylori*, *Proteus* spp., *Serratia* spp., *Campylobacter* spp. are highly antibiotic prevalent in different regions of Bangladesh [10]. This solidifies the perceived fear and indicates the alarming situation of antibiotic resistance in Bangladesh. Despite that, little has been done nationally to identify key factors contributing to antibiotic resistance and the development of policy and regulatory frameworks addressing the issue. This is a concern that must be attended to by the health and administrative authority of Bangladesh, to protect the public health of Bangladeshi citizens which will protect the economy and foster a safe and secure future for the upcoming generations.

## 3 Factors Influencing Antibiotic Resistance in Bangladesh

Several factors contribute to antibiotic misuse in Bangladesh. Studies found that doctors in Bangladesh most often (around 83% of the time) prescribe antibiotics without taking into consideration the clinical tests. The lack of standardized clinical guidelines for antibiotic administration contributes significantly to antibiotic resistance. Antibiotics are often prescribed for minor symptoms, such as those associated with colds and fever, leading patients to discontinue medication prematurely once they feel better. This irrational prescription practice, coupled with the unregulated sale of antibiotics in Bangladesh, fosters widespread self‐medication without consulting healthcare practitioners. A cross‐sectional study in Rajshahi, Bangladesh, revealed that nearly one‐third of participants resorted to self‐medicating with antibiotics for mild symptoms like food poisoning, colds, fever, bowel syndromes, ear infections, and toothaches [11, 12]. A general tendency is prevalent among people to discontinue their antibiotic treatment after the disappearance of signs and symptoms. A study conducted by Hossain et al. reported that around 60% of the study participants discontinued their antibiotic medication without consulting their physicians. This attitude is more prevalent among young people and females. Their findings also suggested that people not finishing their antibiotic medication were at higher risk for acquiring reinfection [13, 14, 15]. Despite the presence of an “Animal Feed Act” that was supposed to prevent antibiotic misuse in Bangladesh, studies revealed in small‐scale poultry farms, antibiotics are used without veterinary supervision and often without prior knowledge about antibiotics by the owner. Many owners use antibiotics as growth supplements, and immunizing agents, some without even knowing a single use of antibiotics [16, 17]. The continuous Antimicrobial Resistance surveillance system is a crucial element for monitoring the current antibiotic resistance of different bacteria and detecting unusual curves in the antibiotic resistance status. This can prepare the responsible authority to take preparation and respond effectively if required. However, evidence suggests, that only 6 out of 64 districts in Bangladesh currently have antibiotic resistance data [10]. Different studies conducted on a subset of the population in Bangladesh, such as poultry farmers, university students, and parents of school‐going children, revealed majority of them lacked good knowledge, attitude, and practices regarding antibiotics. This is a direct contributing factor to the existing antibiotic resistance problem in Bangladesh [18, 19, 20].

## 4 Preventive Measures for Antibiotic Resistance in Bangladesh

Raising awareness and educating physicians on appropriate antibiotic use is a crucial step for tackling the issue which requires a comprehensive approach. For communities, mass media campaigns, school programs, and community health initiatives can disseminate information on the dangers of misuse and resistance. Pharmacy‐based education and behavioral change campaigns can encourage responsible use. For physicians, continuing medical education, updated prescribing guidelines, and monitoring systems can promote evidence‐based practices. Hospitals can establish antimicrobial stewardship teams and facilitate peer learning. Collaborative efforts between public health agencies, medical associations, and policymakers can regulate antibiotic sales and improve diagnostic access. These strategies, supported by regular evaluation, can effectively address antibiotic misuse and resistance. Addressing antibiotic resistance requires collaboration across sectors, including health, agriculture, and education. In the health sector, antimicrobial stewardship programs can work alongside public health initiatives to monitor and regulate antibiotic use. The agriculture sector can minimize the use of antibiotics in livestock by promoting alternatives, such as improved animal husbandry practices and vaccines. Education institutions can play a critical role by integrating antibiotic resistance awareness into curricula and conducting community outreach programs. Joint efforts can include multidisciplinary research initiatives to develop sustainable solutions, policy frameworks to regulate antibiotic use across sectors, and public campaigns emphasizing the interconnectedness of human, animal, and environmental health. This “One Health” approach ensures a comprehensive response to antibiotic resistance.

## 5 Policy Recommendations

A comprehensive and strategic approach, combining the stakeholders and enforcing agencies is required to tackle this existing issue. Based on the different guidelines and policy frameworks provided by the authorities such as WHO, and CDC some recommendations to tackle antibiotic resistance in Bangladesh include—Designing a policy that incorporates (i) strategic vision, (ii) participation, (iii) coordination, (iv) sustainability, (v) equity. Implementing—(i) surveillance, (ii) antimicrobial stewardship, (iii) infection prevention and control, (iv) education, (v) public awareness, (vi) medicines regulation, (v) fostering research and development and developing strong monitoring and regulatory framework. This is a vital step for tackling the alarming condition and saving the upcoming generation from the severe consequences of antibiotic resistance.

## 6 Conclusion

In conclusion, antibiotic resistance represents a profound and escalating public health challenge in Bangladesh, one that demands urgent and coordinated action. Despite its far‐reaching implications for healthcare systems, economic resilience, and community well‐being, this issue has not received the attention it deserves. Tackling this crisis requires a multifaceted approach, including the promotion of antimicrobial stewardship, robust surveillance mechanisms, and widespread public education to curb the misuse of antibiotics. It is equally vital to engage policymakers, healthcare professionals, and citizens in a collective effort to confront this growing threat. Addressing antibiotic resistance is not merely a technical or medical challenge—it is a shared responsibility to protect the health and future of our society.

## Author Contributions

**Md Mahadi Hassan:** conceptualization, methodology, writing–original draft, writing–review and editing; supervision, resources. **Noushin Nohor:** conceptualization, methodology, investigation, writing–original draft, writing–review and editing.

## Conflicts of Interest

The authors declare no conflicts of interest.

## Transparency Statement

The lead author Md. Mahadi Hassan affirms that this manuscript is an honest, accurate, and transparent account of the study being reported; that no important aspects of the study have been omitted; and that any discrepancies from the study as planned (and, if relevant, registered) have been explained.

## Data Availability

The authors have nothing to report.
